# Genetic Constraints, Transcriptome Plasticity, and the Evolutionary Response to Climate Change

**DOI:** 10.3389/fgene.2020.538226

**Published:** 2020-09-18

**Authors:** Michael L. Logan, Christian L. Cox

**Affiliations:** ^1^Department of Biology, University of Nevada, Reno, Reno, NV, United States; ^2^Smithsonian Tropical Research Institute, Panama City, Panama; ^3^Department of Biological Sciences and Institute of Environment, Florida International University, Miami, FL, United States

**Keywords:** climate change, contemporary evolution, gene expression, heritability, molecular evolution, phenotypic plasticity, thermal adaptation, transcriptome

## Abstract

*In situ* adaptation to climate change will be critical for the persistence of many ectotherm species due to their relative lack of dispersal capacity. Climate change is causing increases in both the mean and the variance of environmental temperature, each of which may act as agents of selection on different traits. Importantly, these traits may not be heritable or have the capacity to evolve independently from one another. When genetic constraints prevent the “baseline” values of thermal performance traits from evolving rapidly, phenotypic plasticity driven by gene expression might become critical. We review the literature for evidence that thermal performance traits in ectotherms are heritable and have genetic architectures that permit their unconstrained evolution. Next, we examine the relationship between gene expression and both the magnitude and duration of thermal stress. Finally, we identify genes that are likely to be important for adaptation to a changing climate and determine whether they show patterns consistent with thermal adaptation. Although few studies have measured narrow-sense heritabilities of thermal performance traits, current evidence suggests that the end points of thermal reaction norms (tolerance limits) are moderately heritable and have the potential to evolve rapidly. By contrast, performance at intermediate temperatures has substantially lower evolutionary potential. Moreover, evolution in many species appears to be constrained by genetic correlations such that populations can adapt to either increases in mean temperature or temperature variability, but not both. Finally, many species have the capacity for plastic expression of the transcriptome in response to temperature shifts, with the number of differentially expressed genes increasing with the magnitude, but not the duration, of thermal stress. We use these observations to develop a conceptual model that describes the likely trajectory of genome evolution in response to changes in environmental temperature. Our results indicate that extreme weather events, rather than gradual increases in mean temperature, are more likely to drive genetic and phenotypic change in wild ectotherms.

## Climate Change as an Agent of Selection

The majority of species are dispersal-limited and must adapt to climate change *in situ* if they are to avoid extinction (Hoffmann and Sgro, 2011). The first response of many ectothermic animals will be to adjust their behavior to reduce exposure to stressful temperatures (Kearney et al., 2009; Logan et al., 2013, 2015; Cox et al., 2018; Fey et al., 2019). Nevertheless, behavioral adjustments on their own may be insufficient to maintain fitness, requiring populations to track shifting fitness optima through genetic adaptation and phenotypic plasticity (Berger et al., 2013; Logan et al., 2014, 2019; Buckley et al., 2015; Geerts et al., 2015). A major question that remains is whether populations have heritable variation in climate-related traits such that they may adapt to environmental change over short time scales (Leal and Gunderson, 2012; Walters et al., 2012).

**FIGURE 1:**
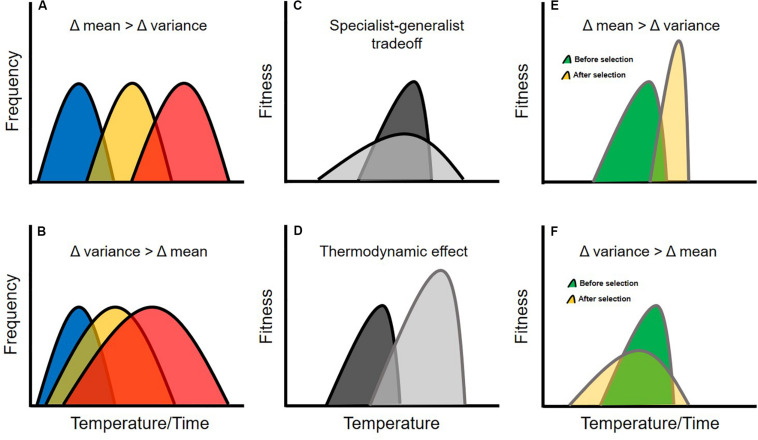
Genetic correlations can constrain the evolution of thermal performance curves, and evolutionary trajectories will likely depend on the specific nature of environmental change. For example, different moments of the environmental temperature distribution can change at different rates, with mean temperature increasing faster than the variance **(A)**, or vise-versa **(B)**. Traits may be constrained in their evolution via a specialist-generalist tradeoff **(C)** which occurs when maximal performance is negatively genetically correlated with performance breadth, or a thermodynamic effect **(D)**, which occurs when maximal performance is positively genetically correlated with the thermal optimum. If these evolutionary constraints occur in the same population, complex evolutionary dynamics can result from selection on thermal performance traits. For example, if mean environmental temperature increases faster than the variance **(E)**, selection should favor an increase in the thermal optimum, with maximal performance also increasing as an indirect result of the thermodynamic effect. As maximal performance increases, performance breadth should then decline as an indirect result of a specialist-generalist tradeoff. Thus, the population becomes well-adapted to mean temperature and maladapted to temperature variability. If the variance of environmental temperature increases faster than the mean **(F)**, selection should favor an increase in performance breadth, with maximal performance decreasing as an indirect result of a specialist-generalist tradeoff. As maximal performance decreases, the thermal optimum should then decline as an indirect result of the thermodynamic effect. Thus, the population becomes well-adapted to temperature variability and maladapted to mean temperature. The colors of the curves in this figure are arbitrary and meant to help increase readability.

Historical data and climate forecasts suggest that shifts in environmental temperature associated with climate change has occurred (and will continue to occur) along two distinct axes (Alley, 2000; IPCC, 2013). First, mean temperature is increasing, primarily as a result of days and seasons that are gradually warming (Figure 1A). Second, the variance of environmental temperature is increasing, primarily because of a rise in the frequency of extreme weather events such as heat waves and cold snaps (Figure 1B). These two axes of thermal change are likely to generate selection on different components of thermal reaction norms (Gabriel and Lynch, 1992; Gilchrist, 1995; Angilletta, 2009). For example, gradual increases in mean temperature will favor genotypes that confer higher thermal optima for ecologically important activities (e.g., genotypes associated with the ability to digest food more effectively at warmer temperatures; Fontaine et al., 2018). By contrast, increases in temperature variability will favor genotypes that boost phenotypic plasticity or whose fitness values are insensitive to temperature (Lynch and Gabriel, 1987; Gabriel and Lynch, 1992).

## The Evolutionary Potential of the Thermal Niche

**FIGURE 2:**
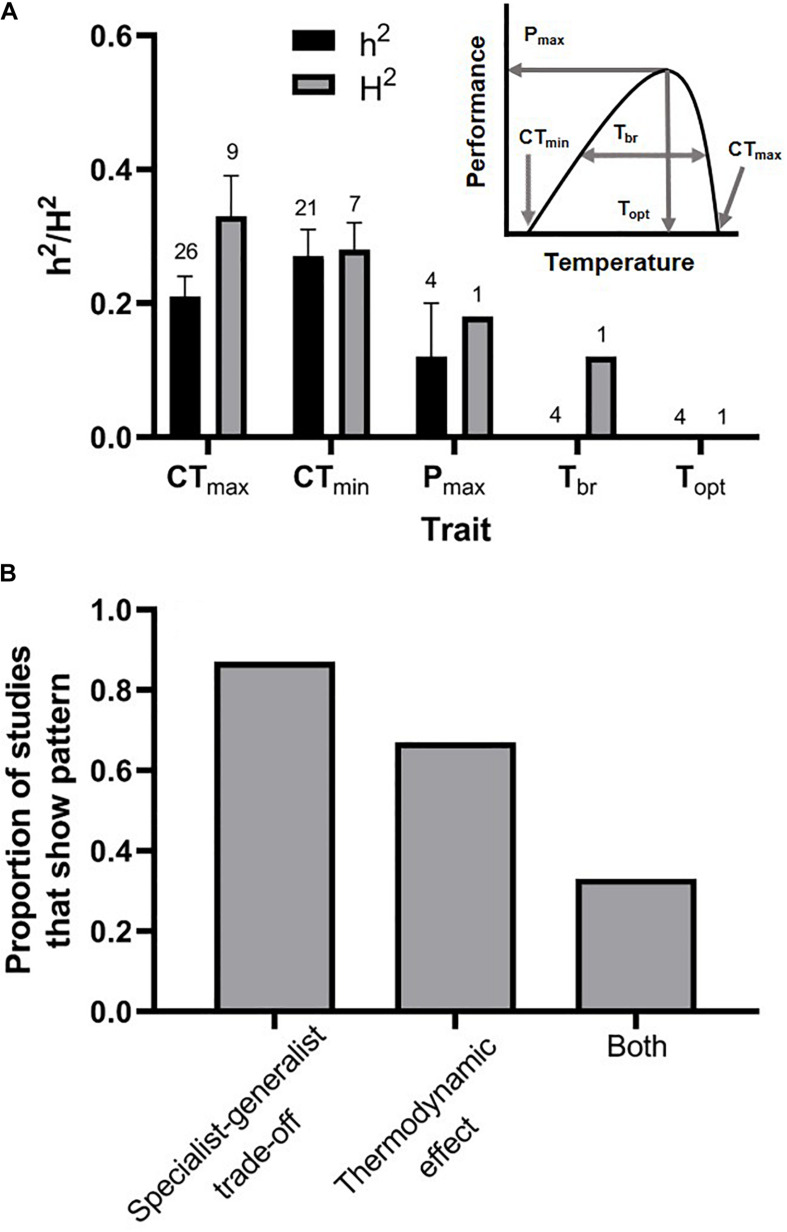
Patterns of genetic constraint on the thermal performance traits that combine to define the shape of thermal performance curves (**A**, inset). **(A)** The critical thermal limits (CT_min_ and CT_max_) are moderately heritable (h^2^ = narrow-sense heritability; H^2^ = broad-sense heritability), whereas the limited evidence that is available suggests that performance breadth (T_br_) and the thermal optimum (T_opt_) lack the capacity to respond rapidly to selection (error bars represent standard errors, and samples sizes are above each bar). **(B)** The majority of studies that tested for either a specialist-generalist tradeoff or a thermodynamic effect underlying the evolution of thermal performance curves found evidence for either one or the other pattern. Two of six studies that tested for both types of constraints in the same population found evidence suggesting that both were operating. Supplementary Table S1 contains the list of studies from which we extracted the values included in this figure.

While theory indicates that the mean or variance of environmental temperature should select for changes in different thermal performance traits, these traits will not evolve unless they are heritable and unconstrained by genetic correlations (Lande and Arnold, 1983; Lynch and Walsh, 1998). In practice, the thermal niche of a given population is usually approximated with a “thermal performance curve” (TPC; Figure 2A, inset). TPCs relate a fitness-proxy (usually an ecologically relevant trait such as locomotor performance) to body temperature (Huey and Hertz, 1984), and often follow an archetypical shape whereby performance increases with body temperature to some optimum (T_opt_) and then sharply declines above that optimum (a pattern driven by the thermodynamics of enzyme function; Hochachka and Somero, 2002). The thermal optimum is expected to be under selection primarily as a result of gradually increasing mean temperatures (Logan et al., 2014). The ends of the TPC (where performance drops to zero) are referred to as the critical thermal limits (critical thermal minimum = CT_min_; critical thermal maximum = CT_max_), and these are closely related to the breadth of the TPC (T_br_). The performance breadth and critical thermal limits are thought to be under selection primarily as a result of changes in the variance of environmental temperature, although performance breadth is probably also affected by selection for changes in performance at intermediate temperatures (Lynch and Gabriel, 1987; Gabriel and Lynch, 1992; Logan et al., 2014). Finally, the height of the TPC describes the maximal performance capacity (P_max_) of the population. These five components of thermal performance curves can be thought of as “thermal performance traits” that combine to define the shape of the thermal niche and may or may not have the capacity to evolve independently of one another (Gomulkiewicz and Kirkpatrick, 1992; Stinchcombe and Kirkpatrick, 2012; Martins et al., 2018; Logan et al., 2020).

Indeed, studies of thermal performance curves across environmental gradients suggest that their shapes may be constrained (Knies et al., 2009; Angilletta et al., 2010; Logan et al., 2013; Phillips et al., 2014). For example, when measured at the level of the phenotype, the area under the curve tends to remain constant even as the shape of the curve changes (Gilchrist, 1996; Kingsolver and Gomulkiewicz, 2003; Izem and Kingsolver, 2005; Phillips et al., 2014). This represents a “specialist-generalist tradeoff” whereby a species can either perform well over a narrow range of temperatures or poorly over a broad range of temperatures (Figure 1C). Specialist-generalist tradeoffs arise from the inability of organisms to optimize biochemical performance across a broad range of temperatures at the subcellular level and often manifest as a negative correlation between whole-organism performance breadth and maximal performance (or as a positive correlation between the critical thermal limits; Hochachka and Somero, 2002). Another pattern commonly observed at the phenotypic level is the “thermodynamic effect” (also referred to as the “hotter-is-better” hypothesis; Angilletta et al., 2010). This effect occurs because biochemical reactions are typically more efficient at warmer temperatures (Hochachka and Somero, 2002), and leads to a positive correlation between the thermal optimum and maximal performance at the whole-organism level (Figure 1D).

If both the specialist-generalist tradeoff and the thermodynamic effect are driven by underlying genetic correlations and occur in the same populations, they represent true evolutionary constraints that can give rise to non-intuitive evolutionary dynamics depending on whether average thermal conditions or extreme weather events are more important sources of selection. For example, if the mean environmental temperature changes faster than the variance, selection should first favor an increase in the thermal optimum, which should then indirectly cause an increase in maximal performance via the thermodynamic effect. This increase in maximal performance should then drive a decrease in performance breadth as a result of a specialist-generalist tradeoff. Thus, adaptation to higher mean temperature can lead to maladaptation with respect to temperature variability (Figure 1E). Alternatively, if the variance in environmental temperature increases faster than the mean, selection should first favor an increase in performance breadth which should indirectly cause a decrease in maximal performance as a result of a specialist-generalist tradeoff. This decrease in maximal performance would then result in a decline in the thermal optimum due to the thermodynamic effect. In this case, adaptation to temperature variability will lead to maladaptation with respect to mean temperature (Figure 1F). Clearly, understanding the extent to which thermal niche evolution is constrained by genetic correlations is critical for generating accurate climate-impact forecasts.

To understand genetic constraints underlying the evolution of the thermal niche, we canvassed the literature for primary, peer-reviewed studies reporting heritabilities (broad and narrow-sense) and genetic correlations underlying the thermal performance traits that make up the thermal niches of animals. We searched the terms “quantitative genetics AND thermal physiology,” “genetic correlations AND thermal physiology,” “heritability AND thermal trait,” “genetics AND specialist-generalist AND temperature,” “genetics AND hotter-is-better,” “genetics AND thermodynamic effect,” “heritability of CTmax,” “heritability of CTmin,” “heritability of thermal optimum,” “heritability AND cold tolerance,” and “heritability AND chill-coma” in Google Scholar in October 2019. Due to the rapid decline of relevant studies after the first few pages of search results, we focused on the first 50 results for each set of search terms (ordered by relevance). To ensure that our sampling was robust, we subsequently (July 2020) included an additional 50 search results on Google Scholar (total = 100 results per search) and conducted a separate set of searches with the same search terms in Thompson Web of Science, again ordered by relevance. In total, we examined more than 1400 results from these databases for possible heritability and genetic correlation estimates. Finally, we included additional studies that we were aware of but that did not come up in our literature searches. These various search avenues likely uncovered the majority of quantitative genetic parameter estimates for our target traits that were available in the literature. Our full database contained 98 independent heritability and genetic correlation estimates from 55 studies. Note that the temperature ramping rates used in these studies varied by several orders of magnitude, and ramping rate is known to affect heritability estimates (Terblanche et al., 2007; Chown et al., 2009). Namely, slow-ramping protocols tend to produce lower heritability estimates, and there is evidence from simulation studies that this may be due to error introduced during longer ramping procedures (Rezende et al., 2011; Santos et al., 2011, 2012). Thus, while most of the heritability estimates included in our analyses were taken from ramping protocols of some kind, when the heritability from both “slow” and “fast” ramping protocols were reported for the same population or species (total of four studies), we only included the latter in our analyses to eliminate pseudoreplication and reduce error as much as possible. For a detailed explanation of how we collated and assessed data from these papers, please see “Extended Methods” in the Online Supplementary Information. We have uploaded the full list of studies included in our analyses of trait heritability and genetic correlations in an online supplementary data file (Supplementary Table S1).

Of the five thermal performance traits that define the shape of the thermal performance curve (Figure 2A, inset), only the critical thermal limits (CT_min_ and CT_max_) were consistently and substantially heritable (Figure 2A). The average broad and narrow-sense heritabilities of CT_min_ were 0.27 and 0.28, respectively. The average broad and narrow-sense heritabilities of CT_max_ were 0.33 and 0.21, respectively. It is interesting to note that phylogenetic studies on some taxa have led to the conclusion that upper thermal limits, but not lower thermal limits, are evolutionarily conserved (Araújo et al., 2013; Grigg and Buckley, 2013; Diamond and Chick, 2017), and this appears to conflict with the relatively high heritability of upper thermal limits observed in controlled breeding studies. The resolution of this conflict may arise from the fact that many species behaviorally thermoregulate during the hottest times of the day or during heat waves, leading to a reduction in the strength of selection on upper thermal tolerance (Muñoz et al., 2014). Thus, even though upper thermal tolerance may be infrequently exposed to selection, this trait may retain its ability to respond to selection in many populations. Indeed, laboratory evolution experiments that expose organisms to selection in warmer environments frequently demonstrate rapid evolutionary change in upper thermal limits (Bettencourt et al., 1999; Gilchrist and Huey, 1999; Sambucetti et al., 2010; Hangartner and Hoffmann, 2016; but see Schou et al., 2014).

To our knowledge, there are only five estimates (from four studies) of the quantitative genetic parameters underlying the other major thermal performance traits: maximal performance, performance breadth, and the thermal optimum. Maximal performance was moderately heritable at an average narrow-sense heritability of 0.12. Every study that examined the performance breadth and the thermal optimum found zero additive genetic variation underlying these traits. Due to the low sample sizes for most of these traits, we did not conduct formal statistical comparisons. Of the studies (*N* = 15) that tested for genetic correlations corresponding to either a specialist-generalist tradeoff or a thermodynamic effect, the majority found evidence of one or the other. 87% of studies found evidence of a specialist-generalist tradeoff, while 67% of studies found evidence of a thermodynamic effect (Figure 2B). Additionally, of the six studies that tested for both a specialist-generalist tradeoff and thermodynamic effect in the same population, two of those studies detected both patterns (Figure 2B). All else remaining equal, these results suggest that the endpoints of the thermal niche (the critical thermal limits) can respond relatively rapidly to selection, although they are likely constrained to some extent by genetic correlations. By contrast, the traits which describe performance at intermediate temperatures (e.g., T_opt_) appear to have minimal capacity for rapid evolution.

## Gene Expression Plasticity

For most organisms, thermal performance traits are not fixed across environmental conditions, but instead can exhibit adaptive or non-adaptive phenotypic plasticity (Scheiner, 1993; Via et al., 1995; Ghalambor et al., 2007, 2015). For example, previous exposure to cool temperatures reduced the recovery time after induction of chill- coma in fruit flies (*Drosophila melanogaster*) compared to flies reared at intermediate temperatures (Ayrinhac et al., 2004). Similarly, acclimation to warmer temperatures increased time to immobilization (a measure of heat tolerance) in the freshwater crustacean *Daphnia magna* (Yampolsky et al., 2014a).

The mechanism driving most phenotypic plasticity is changes in gene expression (Scheiner, 1993; Schlichting and Pigliucci, 1993; Schlichting and Smith, 2002; Chen et al., 2017). Shifts in gene expression can involve only a few genes (Hamdoun et al., 2003), or can occur across the entire transcriptome (Bay and Palumbi, 2015). For example, shifts in the expression of genes in the heat-shock protein (*hsp*) 70 family seem to underlie phenotypic plasticity in thermal tolerance limits in the oyster *Crassostrea gigas* (Hamdoun et al., 2003), whereas exposure to warm temperatures was associated with alterations of whole-transcriptome expression and increased heat tolerance in the coral *Acropora nana* (Bay and Palumbi, 2015). Broadly, this suggests that phenotypic plasticity, mediated by gene expression, is important for the adaptive response to global climate change.

To understand how gene expression might be involved in the response to climate change, we canvassed the literature for studies that measured transcriptomic responses to thermal stress in ectothermic animals. We searched the terms “transcriptome heat stress,” “transcriptome expression temperature vertebrate,” “gene expression heat vertebrate,” “transcriptome expression thermal,” “transcriptome thermal,” and “gene expression thermal” in Google Scholar during October 2019. We conducted a subsequent, deeper search (100 results for each set of search terms) in both Google Scholar and Thompson Web of Science during July 2020. These queries returned hundreds of journal articles, each of which we evaluated for relevance. Ultimately, this process yielded 36 articles containing 42 independent estimates of the effects of temperature on the transcriptomic response in ectotherms. These studies spanned early microarray work to recent experiments that leveraged high-throughput RNA sequencing, and they focused on acute, reversible gene expression responses rather than fixed changes that may occur over development (Table 1).

**TABLE 1:** Studies of transcriptomic responses to temperature change.

Study	Organism	Species	Data type	Change in transcriptome expression?	Altered *hsp* expression?
Akashi et al. (2016)	Lizard	*Anolis allogus*	RNAseq	Y	Y
Akashi et al. (2016)	Lizard	*Anolis homolechis*	RNAseq	Y	Y
Akashi et al. (2016)	Lizard	*Anolis sagrei*	RNAseq	Y	Y
Coughlin et al. (2019)	Fish	*Osmerus mordax*	RNASEq	Y	Y
Cui et al. (2019)	Insect	*Megacopta cribaria*	RNAseq	Y	Y
Etges et al. (2017)	Insect	*Drosophila mojavensis*	RNAseq	Y	Y
Gleason and Burton (2015)	Mollusc	*Chlorostoma funebralis*	RNAseq	Y	Y
Gracey et al. (2004)	Fish	*Cyprinus carpio*	Microarray	Y	Y
Hu et al. (2016)	Fish	*Danio rerio*	RNAseq	Y	N/A
Hu et al. (2016)	Fish	*Oreochromis niloticus*	RNAseq	Y	N/A
Jayasundara et al. (2013)	Fish	*Thunnus orientalis*	Microarray	Y	Y
Jesus et al. (2016)	Fish	*Squalius carolitertii*	RNAseq	Y	Y
Jesus et al. (2016)	Fish	*Squalius torgalensis*	RNAseq	Y	Y
Kassahn et al. (2007)	Fish	*Pomacentrus moluccensis*	Microarray	Y	Y
Kim et al. (2017)	Mollusc	*Crassostrea gigas*	RNAseq	Y	Y
Lewis et al. (2010)	Fish	*Onchorhyncus mykiss*	Microarray	Y	Y
Li et al. (2017)	Fish	*Onchorhyncus mykiss*	RNAseq	Y	Y
Li et al. (2019)	Fish	*Megalobroma amblycephala*	RNAseq	Y	Y
Lim et al. (2016)	Mollusc	*Crassostrea gigas*	RNAseq	Y	Y
Liu et al. (2013)	Fish	*Ictalurus* hybrids	RNAseq	Y	N/A
Lockwood et al. (2010)	Mollusc	*Mytilus trossulus*	Microarray	Y	Y
Lockwood et al. (2010)	Mollusc	*Mytilus galloprovincialis*	Microarray	Y	Y
Logan and Somero (2011)	Fish	*Gillichthys mirabilis*	Microarray	Y	Y
Moskalev et al. (2015)	Insect	*Drosophila melanogaster*	RNAseq	Y	N/A
Moya et al. (2012)	Cnidarian	*Anemonia viridis*	Microarray	Y	Y
Narum and Campbell (2015)	Fish	*Oncorhynchus mykiss*	RNAseq	Y	Y
Qian and Xue (2016)	Fish	*Larimichthys crocea*	RNAseq	Y	Y
Quinn et al. (2011)	Fish	*Salvelinus alpinus*	Microarray	Y	Y
Semmouri et al. (2019)	Crustacean	*Temora longicornis*	RNAseq	Y	Y
Shi et al. (2019)	Fish	*Salmo salar*	RNAseq	Y	Y
Smith et al. (2013)	Fish	*Melanotaenia duboulayi*	RNAseq	Y	Y
Smolina et al. (2015)	Crustacean	*Calanus finmarchius*	RNAseq	Y	Y
Smolina et al. (2015)	Crustacean	*Calanus glacialis*	RNAseq	Y	N/A
Sørensen et al. (2016)	Insect	*Drosophila melanogaster*	RNAseq	Y	Y
Stillman and Tagmount (2009)	Crustacean	*Petrolisthes cinctipes*	Microarray	Y	Y
Vornanen et al. (2005)	Fish	*Onchorhyncus mykiss*	Microarray	Y	Y
Wang et al. (2014)	Mollusc	*Echinolittoria malacaria*	RNAseq	Y	Y
Wellenreuther et al. (2019)	Fish	*Chrysophus auratus*	RNAseq	Y	Y
Xiao et al. (2016)	Spider	*Pardosa pseudoannulata*	RNAseq	Y	Y
Yampolsky et al. (2014b)	Crustacean	*Daphnia* sp.	Microarray	Y	N/A
Yang et al. (2016)	Fish	*Ctenopharyngodon idellus*	RNAseq	Y	Y
Zheng et al. (2019)	Crustacean	*Marsupaenus japonicus*	RNASeq	Y	Y

**Irrespective of the methodology or focal taxon, all experiments detected changes in gene expression when the organism was exposed to a change in temperature. Additionally, all studies that reported analyses of heat-shock protein (hsp) genes detected shifts in the expression of these genes.**

**FIGURE 3:**
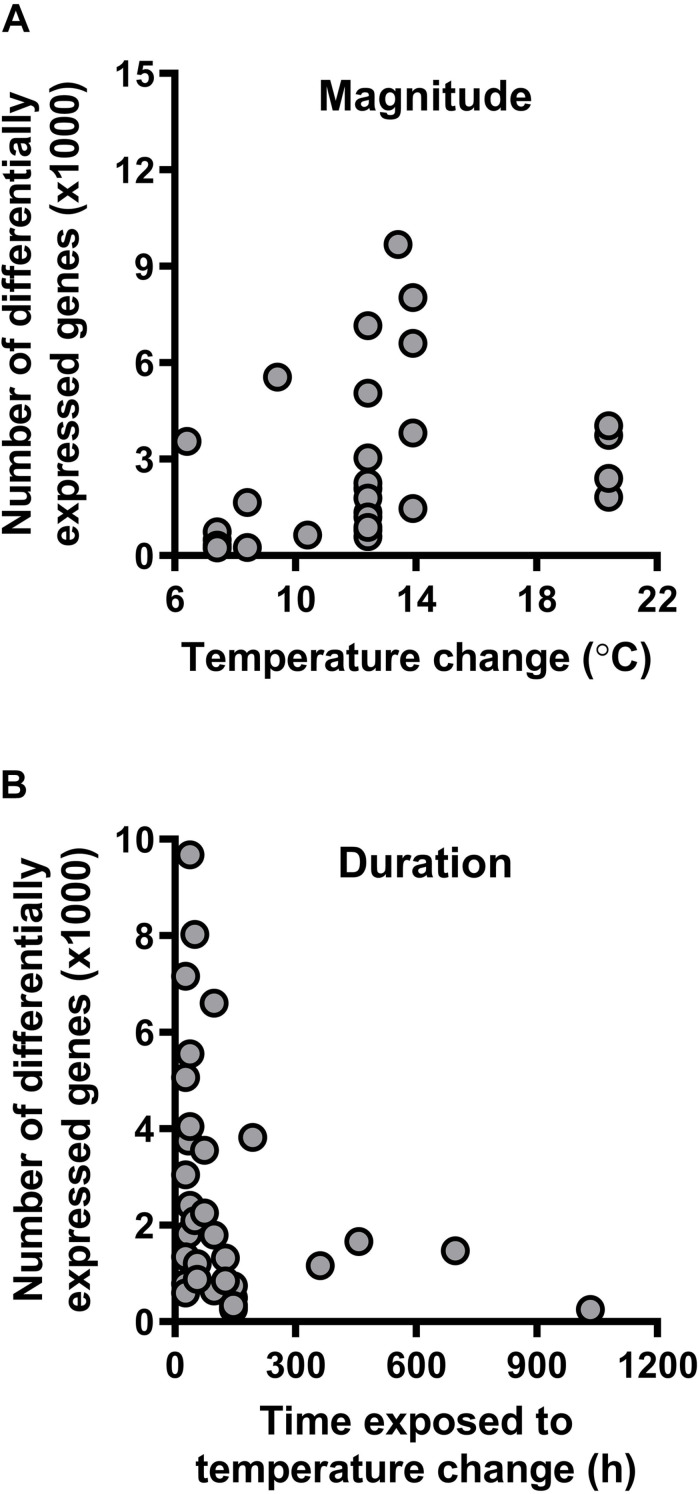
Transcriptomic responses to temperature change. **(A)** The number of differentially expressed genes increases with the magnitude of temperature change to which an organism is exposed. **(B)** The number of differentially expressed genes does not vary with duration of exposure to a given magnitude of temperature change. See Table 1 for the list of studies from which we extracted the values included in this figure.

All species in these experiments, which range from arthropods to vertebrates and occur in diverse habitats across the globe, shift expression of their transcriptome in response to thermal changes (Table 1). However, the temperature changes experienced by organisms in these studies varied greatly in their magnitude and duration. When restricting the analyses to RNAseq studies and excluding whole-organism studies (Supplementary Table S2), we found that the magnitude of temperature change [*F*_(3, 33)_ = 13.0448, *P* = 0.0010; Figure 3A], but not the duration of exposure [*F*_(3, 33)_ = 2.1269, *P* = 0.1542: Figure 3B] predicted the number of log-transformed differentially expressed genes when controlling for log-transformed transcriptome size [*F*_(3_,_33)_ = 3.3718, *P* = 0.0753] using linear regression models. These results indicate that brief, severe weather events could impact gene expression and phenotypic plasticity more profoundly than longer-term changes in thermal conditions.

A previous study by Gunderson and Stillman (2015) reported limited potential for plastic responses to warming across a broad range of organisms. This suggests that there may not be a one-to-one correspondence between the magnitude of gene expression shifts and physiological plasticity in the typical whole-organism traits that investigators measure (e.g., CT_max_ and CT_min_). Indeed, not all mRNAs that are transcribed will be translated into proteins (Liu et al., 2016), possibly leading to a discordance between the magnitude of gene expression plasticity and phenotypic plasticity. Additionally, many of the studies cited in Gunderson and Stillman (2015) involved ramping or constant-exposure thermal stress experiments, which may be less likely to result in large-scale changes in gene expression. In general, further work is needed to understand the link between gene expression plasticity under large magnitude shifts in temperature and phenotypic plasticity in thermal tolerance limits.

Among the genes that were differentially expressed in response to temperature, gene ontology (and similar) analyses have found that biological processes associated with protein synthesis, folding and degradation, oxygen transport, and biological and cellular responses to heat and other stress-stimuli are often significantly enriched (Supplementary Table S3). Heat shock proteins, which are a conserved set of molecular chaperone proteins with important roles for responding to stress in general, and heat stress in particular (Feder and Hofmann, 1999), were especially important. Genes for heat-shock proteins were frequently (94% of species, Table 1) affected by changes in temperature, with shifts in expression often occurring in well-characterized canonical genes such as hsp40 (or DNAJ), hsp70, and hsp90 (Supplementary Table S4). Because expression of heat shock proteins is usually altered in response to changing temperature and has been linked to phenotypic plasticity (Hamdoun et al., 2003), these proteins are likely to be important targets of selection as global climate change progresses.

## How Will Genomes Respond to Selection When Thermal Environments Shift?

Rapid environmental change can induce selection on the genome in two major ways. First, selection can target sequence variation in crucial protein-coding genes (Hoekstra et al., 2004; Rosenblum et al., 2010). This is most likely to occur when the capacity for gene expression plasticity is minimal or under weak selection and may manifest as changes in loci that affect the “baseline” values of thermal performance traits like the thermal optimum or the critical thermal limits. Second, if variation among individuals in gene expression plasticity is high or under strong selection, the primary adaptive response to a changing climate may be shifts in loci that are associated with variation in gene expression (Behera and Nanjundiah, 1995; Ghalambor et al., 2015; Campbell-Staton et al., 2020). Selection on gene expression could target trans-regulatory pathways or the upstream and downstream cis-regulatory regions that affect expression of individual genes (Schlichting and Pigliucci, 1993; Via, 1993; Campbell-Staton et al., 2020), and is likely to increase the frequency of genotypes with broad phenotypic reaction norms. Alternatively, selection could target genes that regulate epigenetic mechanisms such as histone modification or methylation (Johannes et al., 2009; Furrow and Feldman, 2014).

**FIGURE 4:**
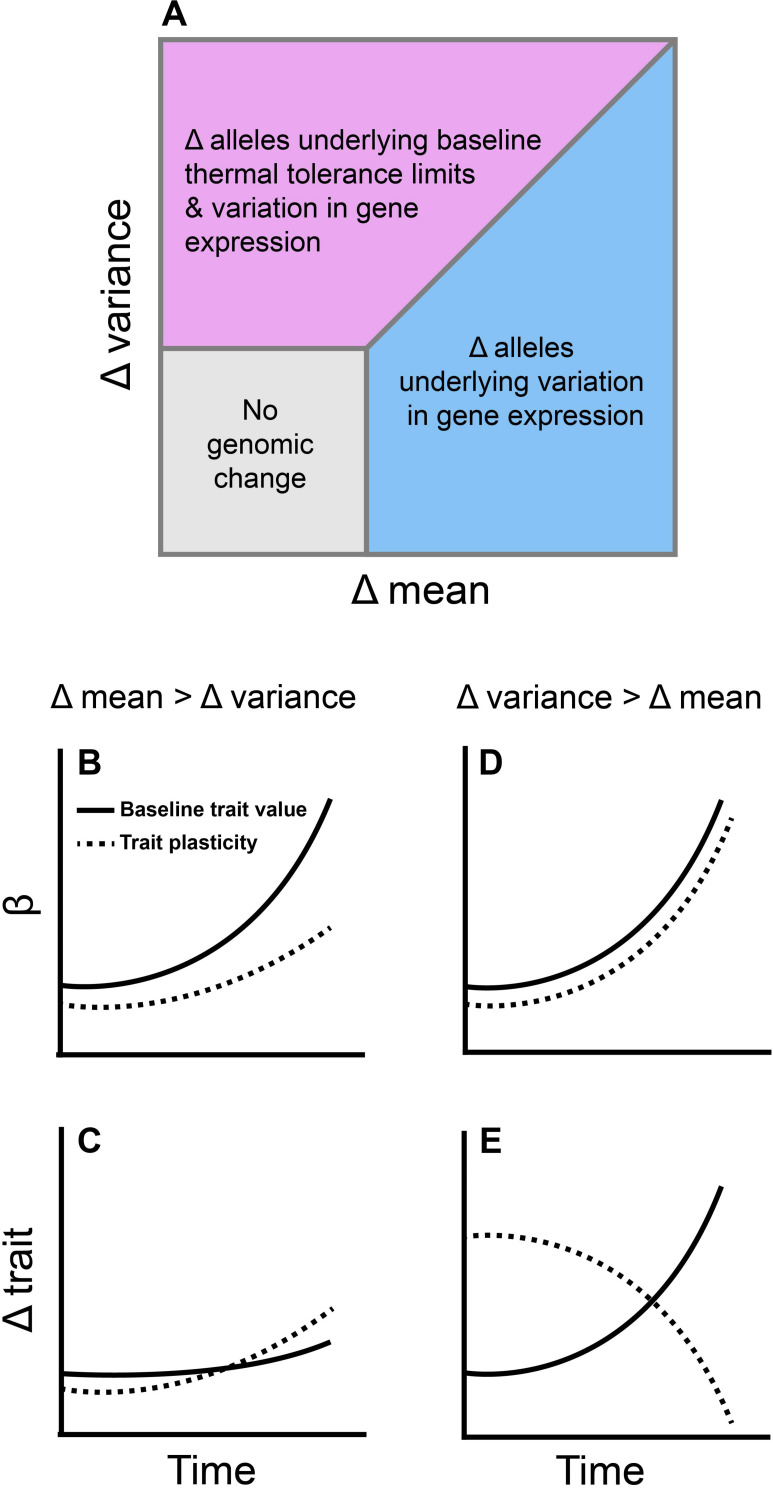
The rates at which different moments of the environmental temperature distribution change are likely to impact observed patterns of genomic and phenotypic evolution. **(A)** Based on patterns of genetic variation reported in the literature, alleles underlying variation in gene expression (blue and purple regions) are more likely to change in frequency during environmental change than alleles underlying baseline thermal tolerance. Only in cases where the change in the variance of temperature is equal to or higher than the change in mean temperature should alleles associated with baseline trait values shift substantially (purple region). **(B)** If mean environmental temperature changes faster than the variance, selection (β) should favor an increase in baseline values of traits like the thermal optimum, while selection for increased plasticity should gradually rise as baseline values fail to evolve due to genetic constraints. **(C)** When mean environmental temperature increases faster than the variance, traits that correspond to performance at intermediate temperatures (such as the thermal optimum) should evolve slowly while plasticity increases to compensate for the lack of heritability in the baseline values of these traits. **(D)** When the variance of environmental temperature increases faster than the mean, selection should favor an increase in both the baseline values of traits which correspond to performance at extreme temperatures (e.g., the critical thermal limits) and the plasticity of such traits. **(E)** Because the critical thermal limits are heritable in most species, they should evolve in response to selection. This should lead to a reduction in the rate of change in plasticity as baseline trait values become locally adapted. Note that this assumes more genetic variation is initially present in baseline thermal tolerance than in its plasticity. The dashed and solid lines in **(E)** would be flipped if there was more genetic variation underlying the plasticity of thermal tolerance than in their baseline values.

Our review of the literature suggests several pathways by which shifts in environmental temperature distributions should impact genomic variation (Figure 4). To date, studies suggest that the endpoints of the thermal niche (the critical thermal limits) are heritable, whereas performance at intermediate temperatures (e.g., T_opt_) are not (Figure 2). The critical thermal limits are most important under extreme weather conditions such as heat waves and cold snaps (Campbell-Staton et al., 2017), indicating that baseline genetic variation for thermal performance may be more capable of responding to these extreme events than to gradual changes in mean temperature (although adaptation to extreme weather events may still be constrained by genetic correlations; Figures 1, 2). Similarly, most species appear to alter gene expression when they are exposed to short-term shifts in temperature (Table 1), and the number of genes that are differentially expressed increases with the magnitude of the temperature shift (akin to a short-term extreme weather event; Figure 3A). In contrast, the number of differentially expressed genes did not vary with duration of exposure to these temperature shifts (Figure 3B). This pattern may reflect a reduced importance of gene expression plasticity when environmental change is dominated by longer-term increases in mean temperature. Taken together, these data suggest that genomic responses will be more rapid and pronounced in response to changes in the frequency of extreme weather events than in response to gradual warming.

Emerging patterns from genomic and transcriptomic studies also suggest that the specific nature of environmental change will be important for determining trajectories of molecular evolution (Figure 4). As environmental temperature distributions change, different moments of the distribution can shift at different rates. Because the mean and variance of environmental temperatures drive selection on separate traits that have varying levels of additive genetic variation underlying them, we would expect “baseline” genetic adaptation and changes in gene expression plasticity to make up different components of the adaptive response depending on the details of environmental change (Figure 4A). If both the change in mean temperature and the change in variance are low, then selection will be weak or non-existent on all traits (compensatory responses might be entirely behavioral, for example), leading to zero molecular and phenotypic evolution. However, if the change in mean temperature is higher than the change in variance, we would expect shifts in alleles underlying variation in gene expression instead of shifts in alleles underlying “baseline” values of thermal traits. This is because, even though selection favoring better performance at intermediate temperatures should be high (Figure 4B), we would predict minimal evolution (Figure 4C) since the relevant traits (e.g., T_opt_) appear to lack additive genetic variation (Figure 2A).

Patterns of molecular and phenotypic evolution should be different if the variance of environmental temperature changes faster than the mean. There appears to be substantial genetic variation in both the critical thermal limits and the gene expression response to thermal stress within populations (Figures 2, 3 and Table 1). Thus, selection for performance at extreme temperatures should favor loci that correspond to high and low baseline values of CT_max_ and CT_min_, respectively (Figures 4A,D). Selection for increased gene expression plasticity should also increase in strength as extreme weather events become more common (Figure 4D), but the rate of change in plasticity should decline as baseline trait values become locally adapted (Figure 4E).

## Future Research Directions

The literature on the genomic and transcriptomic basis of thermal adaptation hints at multiple potential evolutionary outcomes depending on the nature of environmental change. Nevertheless, these observations should be considered preliminary, as comparatively few studies have investigated the quantitative genetic basis of full thermal performance curves. Thus, estimates of heritabilities and genetic correlations underlying performance at intermediate temperatures (temperatures at or close to the thermal optimum) are exceedingly rare. To our knowledge, only three studies have estimated narrow-sense heritabilities of the thermal optimum and performance breadth. Two of these were on lizards (Logan et al., 2018; Martins et al., 2018) and the third was on an invasive population of harlequin beetles (Logan et al., 2020). A fourth study reported broad-sense heritabilities of the performance breadth and the thermal optimum in parasitoid wasps (Gilchrist, 1996). Although a general pattern of low genetic variation in these traits is starting to emerge from this research, we need many more studies of the quantitative genetics of full thermal performance curves to understand whether performance at intermediate temperatures truly lacks rapid evolutionary potential, or whether the patterns we report here are an artifact of insufficient sampling.

Most studies have examined genetic variation in either the baseline values of thermal traits or their plasticity, but rarely both. Future work should focus on the genetic basis of baseline values of thermal traits and their plasticity in the same populations to tease apart the independent contribution of both to local adaptation under environmental change. A rare example of such a study is Gerken et al. (2015), who assessed the heritability and genomic basis of both basal cold tolerance and its plasticity in laboratory lines of fruit flies. They found that baseline thermal tolerance was genetically correlated with its plasticity, implying that adaptation is constrained when both the mean and variance of temperature are increasing.

Our review suggests that genes in the heat shock protein family are a likely target for selection when environments first shift, and the evolutionary potential of these genes may be a major determinant of populations’ resilience in the face of climate change. Past evolution of heat shock proteins is dominated by repeated duplications and insertion events, which might have been followed by neofunctionalization (Waters, 1995; Franck et al., 2004; Yamashita et al., 2004; Huang et al., 2008). At least in some contexts, there is evidence of directional selection on heat-shock proteins (Bettencourt et al., 2002; Fares et al., 2002). However, we do not know whether selection acts primarily on the coding sequences of these genes or on their upstream and downstream regulatory regions. Future work should determine the level of functional sequence variation underlying this family of genes in wild populations, and the relationship between heat-shock protein evolution and population mean fitness.

Our results suggest that the evolution of gene expression plasticity may be particularly important in maintaining fitness under climate change, not only because a number of thermal traits appear to lack genetic variation in their baseline values, but also because extreme weather events are rising in frequency. Moreover, past research has revealed that the capacity for gene expression plasticity can be heritable and evolve rapidly (Gerken et al., 2015; Leder et al., 2015). Additionally, variation in plasticity that is not genetic may persist across generations due to epigenetic mechanisms and can be important for population persistence in the initial stages of environmental change (Geng et al., 2013; Schlichting and Wund, 2014). Despite growing evidence that the evolution of phenotypic plasticity may be critical for organismal responses to climate change, it is still unclear how selection on plasticity is manifested at the level of the genome. Related questions that should be addressed by future research include 1) If extreme weather events select for higher gene expression plasticity, should we expect fast changes in regulatory regions of the genome, non-coding regions, or both? 2) Does selection for increased phenotypic plasticity constrain the evolution of baseline thermal tolerance (or vice versa)? Additional studies of within-population variation in baseline thermal tolerance and plasticity, and the genetic loci associated with each, are sorely needed.

## Conclusion

Our review suggests that several general rules may be emerging from studies of the genetic and transcriptomic basis of thermal performance:

1. In many species, there is more genetic variation in performance at extremely high or low temperatures than in performance at intermediate temperatures.
2. Gene expression plasticity is rampant when organisms are exposed to acute thermal stress.
3. Patterns (1) and (2) indicate that populations are more likely to evolve rapidly in response to extreme weather events than in response to gradual changes in mean temperature, and the rate at which different moments of the temperature distribution change will determine the dominant trajectory of phenotypic and genetic evolution.
4. Gene regulatory networks linked to heat shock proteins are likely to be a major target of selection as environmental temperatures become warmer and more variable.

Finally, our work highlights the need for further studies on the quantitative genetic basis of thermal performance curves and the interactions between baseline thermal tolerance and gene expression plasticity. Continued advances in this field should lead to substantial improvements in our ability to predict the viability of animal populations as our planet continues to change.

## Author Contributions

Both authors conceived of the study, reviewed the literature, and wrote the manuscript.

## Conflict of Interest

The authors declare that the research was conducted in the absence of any commercial or financial relationships that could be construed as a potential conflict of interest.

## Glossary of Terms

**Table:**

Thermal performance curve:	The mathematical relationship between an ecologically relevant metric of performance (e.g., locomotion, energy assimilation, immune function, etc.) and organismal body temperature. These curves are often used to approximate a populations’ thermal niche and can be sub-divided into “thermal performance traits” that describe different aspects of its shape.
Thermal performance trait:	A phenotypic trait that describes performance (e.g., locomotion, energy assimilation, immune function, etc.) at one or a range of temperatures. These traits combine to describe the shape of a population’s thermal performance curve.
Narrow-sense heritability (h^2^):	The component of phenotypic variation in a trait that is comprised of additive genetic variation. Narrow-sense heritability describes the capacity for a trait to respond efficiently to selection.
Broad-sense heritability (H^2^):	The component of phenotypic variation in a trait that is comprised of both additive and non-additive genetic variation, including the effects of dominance and epistasis. Broad-sense heritability includes forms of genetic variation that do not respond efficiently to selection (e.g., recessive alleles that can remain hidden from selection in the heterozygous state).
Genetic correlation:	Positive or negative statistical correlation between genes underlying different phenotypic traits. Genetic correlations often arise from linkage disequilibrium or pleiotropy and can cause correlated evolution of a trait that is not itself under direct selection, but rather is genetically correlated with a different trait that is under direct selection.
Gene expression:	Transcription of mRNA from the genome, which can later be translated into a protein. All mRNA transcripts expressed in a cell, tissue, or organism are referred to as the transcriptome.
Gene expression plasticity:	The ability to alter gene expression in response to an environmental cue. This could be measured at the level of the organism (i.e., the total number of genes that shift their expression) or at the level of an individual gene (i.e., the number and persistence of gene transcripts).
Phenotypic plasticity:	The capacity of the same genotype to produce different phenotypes in different environments. The functional basis of phenotypic plasticity is usually gene expression plasticity.
