# Supplementary material for: Genetic Constraints, Transcriptome Plasticity, and the Evolutionary Response to Climate Change
Source: Front Genet. 2020 Sep 18;11:538226. doi: 10.3389/fgene.2020.538226 (PMC7531272; doi:10.3389/fgene.2020.538226)
Supplement: Supplementary file 1 [file Data_Sheet_1.ZIP › Extended Methods.docx]

**Supplementary Material**

**Extended Methods**

To understand genetic constraints underlying the evolution of the thermal niche, we canvassed the literature for studies reporting heritabilities (broad and narrow-sense) and genetic correlations underlying the thermal performance traits that make up the thermal niches of animals. Methods and experimental design varied widely among studies. We applied the following rules for evaluating heritabilities and genetic correlations:

1. Estimates of heritability and genetic correlations from different populations of the same species were included as separate values.
2. If studies reported heritability estimates from multiple genetic lines originating from the same population, we averaged the estimates across lines.
3. If studies reported separate estimates for males and females, or for different life stages, we averaged the estimates across sexes or life stages.
4. If studies reported results from different statistical approaches, we averaged the estimates across approaches.
5. If estimates were reported from different experimental approaches (e.g. slow-ramping versus static or fast-ramping assays for thermal tolerance), we only included the approach that we deemed less error-prone. For example, we used estimates from static or fast-ramping, rather than slow-ramping, protocols, if the former were available, since simulation studies have shown that slow ramping protocols systematically underestimate heritabilities of thermal tolerance traits (Rezende et al., 2011; Santos et al., 2011; 2012).
6. If studies estimated the heritability of the same thermal trait across a range of experimental conditions, we averaged the values across conditions, unless the experiment followed (7), below.
7. If an experimental procedure was applied with the expressed goal of changing heritability or genetic correlation values (e.g. a “bottleneck” treatment where the experimenters were attempting to reduce genetic variation), we did not include those estimates in our review.
8. In studies where different assays of heat or cold tolerance (e.g. knockdown versus lethal temperature) were evaluated in the same population, we averaged the heritability estimates from the different assays under the assumption that they combine to give a sense of general heat or cold tolerance for that species or population.
9. For calculating the percentage of studies that found evidence of a specialist-generalist tradeoff or a thermodynamic effect, we included only papers that looked for such effects at the genetic level (via breeding experiments or laboratory evolution studies). We considered a study as finding such effects if they detected at least one incontrovertible line of evidence supporting the effect (e.g. a genetic correlation between the thermal optimum and maximal performance).

**References**

Rezende, E.L., Tejedo, M., and Santos, M. (2011). Estimating the adaptive potential of critical thermal limits: methodological problems and evolutionary implications. *Functional Ecology* 25**,** 111-121.

Santos, M., Castaneda, L.E., and Rezende, E.L. (2011). Making sense of heat tolerance estimates in ectotherms: lessons from Drosophila. *Functional Ecology* 25**,** 1169-1180.

Santos, M., Castaneda, L.E., and Rezende, E.L. (2012). Keeping pace with climate change: what is wrong with the evolutionary potential of upper thermal limits? *Ecology and Evolution* 2**,** 2866-2880.
